# Dietary Macronutrient Intake and Vascular Health in Patients with Long COVID: The BioICOPER Study

**DOI:** 10.3390/nu18122028

**Published:** 2026-06-22

**Authors:** Nuria Suárez-Moreno, Elena Navarro-Matías, Silvia Arroyo-Romero, Alicia Navarro-Cáceres, Andrea Domínguez-Martín, Cristina Lugones-Sanchez, Susana Gonzalez-Sanchez, Manuel A. Gómez-Marcos, Marta Gómez-Sánchez, Leticia Gómez-Sánchez, BioICOPER Investigators Group

**Affiliations:** 1 Primary Care Research Unit of Salamanca (APISAL), Avd. Portugal, 37005 Salamanca, Spain; nuria.suarez@usal.es (N.S.-M.); enavarro@saludcastillayleon.es (E.N.-M.); silvia_ar@usal.es (S.A.-R.); alicia.nav@usal.es (A.N.-C.); andreadm@usal.es (A.D.-M.); cristinals@usal.es (C.L.-S.); gongar04@gmail.com (S.G.-S.); 2 Instituto de Investigación Biomedico de Salamanca (IBSAL), Paseo de San Vicente, 37007 Salamanca, Spain; 3 Departamento de Medicina, Facultad de Medicina, Universidad de Salamanca, 28046 Salamanca, Spain; 4 Servicio de Salud de Castilla y Leon—SACYL, Servicio Regional de Salud, 37005 Salamanca, Spain; 5 Red de Investigación Sobre Cronicidad, Atención Primaria y Promoción de la Salud (RICAPPS), 37005 Salamanca, Spain; 6 Servicio de Hospitalización Domiciliaria, Hospital Universitario Marqués de Valdecilla, 39008 Santander, Spain; martagmzsnchz@gmail.com; 7 Servicio de Urgencias, Hospital Universitario de La Paz, P. de la Castellana, 261, 28046 Madrid, Spain; leticiagmzsnchz@gmail.com

**Keywords:** Long COVID, arterial stiffness, vascular health, macronutrient intake, diet quality and vascular aging

## Abstract

**Background:** Long COVID (LC) has been associated with persistent endothelial dysfunction and vascular impairment. Although nutrition is a key modifiable determinant of cardiovascular health, the relationship between dietary macronutrient intake and vascular alterations in LC remains poorly understood. **Objective:** To evaluate the association between dietary macronutrient intake and markers of vascular structure, arterial stiffness, and vascular aging in patients with LC, including potential sex differences. **Methods:** We conducted a cross-sectional study including 304 patients with LC. Dietary intake was assessed using a validated 7-day dietary record (EVIDENT study). Vascular evaluation included carotid intima–media thickness (cIMT), carotid–femoral pulse wave velocity (cfPWV), brachial–ankle pulse wave velocity (baPWV), cardio-ankle vascular index (CAVI), augmentation index adjusted to a heart rate of 75 beats per minute (AIx@75), and vascular aging index (VAI), measured using carotid ultrasound and validated devices (SphygmoCor^®^ and VaSera^®^). **Results:** The mean age was 53 ± 12, higher in men (*p* = 0.001). The study included 207 women (68%) and 97 men (32%). Energy intake and carbohydrate intake in g/day showed a negative association with cfPWV in Model 2 (energy intake: β = −0.06; 95% CI: −0.11 to −0.01; *p* = 0.02; carbohydrate intake: β = −0.47; 95% CI: −0.87 to −0.07; *p* = 0.02). The percentage of carbohydrate/total energy intake was positively associated with AIx@75 in Model 2 (β = 0.8; 95% CI 0.12 to 1.49; *p* = 0.02), and percentage of fat/total energy intake showed a consistent inverse association (β = −0.30; 95% CI: −0.49 to −0.11; *p* = 0.002). No significant associations were observed for cIMT, baPWV, CAVI or VAI. **Conclusions:** In patients with LC, total energy intake and absolute carbohydrate intake were negatively associated with cfPWV, whereas the relative contribution of carbohydrates and fats to total energy intake showed divergent associations with AIx@75. These findings suggest that both absolute macronutrient intake and relative macronutrient distribution may be related to central arterial stiffness and wave reflection parameters LC. However, given the cross-sectional design of the study, these results should be interpreted as exploratory and do not allow causal inference. Further longitudinal and interventional studies are needed to confirm these findings and to assess whether nutritional strategies may contribute to modulating vascular risk in this population.

## 1. Introduction

Long COVID syndrome (LC) represents an important global public health concern, given its frequent occurrence and considerable burden on patients’ quality of life [1,2,3]. Current evidence supports a marked vascular tropism of SARS-CoV-2 during COVID-19, leading to persistent endothelial dysfunction and a chronic pro-inflammatory state [4,5]. Clinically, this endothelial impairment contributes to increased arterial stiffness and accelerated vascular aging, which may manifest months or even years after the acute COVID-19 episode [4,6]. Moreover, the effects on vascular function may vary by sex, as women with LC have been reported to show worse arterial elasticity [7]. Since arterial stiffness independently predicts cardiovascular morbidity and mortality, reducing vascular impairment has become a major therapeutic goal in individuals with LC [8,9,10,11,12,13].

Nutrition plays a fundamental role in cardiovascular prevention and represents a modifiable factor closely linked to vascular function. In the general population, growing evidence indicates that both macronutrient quantity and quality are related to arterial structure and function, underscoring the potential relevance of dietary composition for vascular integrity [14,15]. Dietary composition plays a critical role: high intake of refined carbohydrates, simple sugars, and saturated fats has been associated with increased systemic inflammation and endothelial dysfunction [16,17,18], whereas dietary fiber and high-quality dietary patterns exert protective vascular effects [15,19]. In this context, recent studies published in Nutrients highlight the role of diet quality and macronutrient balance in modulating vascular stiffness and cardiometabolic risk [14,19,20]. However, despite this growing body of evidence, the specific role of macronutrient intake in modulating arterial stiffness and vascular aging in individuals with LC remains poorly understood.

Addressing this knowledge gap requires the integration of robust dietary assessment tools with comprehensive, non-invasive vascular measurements across the arterial tree. The use of validated dietary assessment instruments, such as those developed within the EVIDENT study [21], combined with multimodal vascular evaluation—including carotid intima–media thickness (cIMT) as a marker of vascular structure, carotid–femoral pulse wave velocity (cfPWV) for central arterial stiffness, brachial–ankle pulse wave velocity (baPWV) for peripheral stiffness, and the cardio-ankle vascular index (CAVI), which provides a blood pressure–independent assessment of arterial stiffness [22]—enables a detailed characterization of vascular status. Additionally, systemic arterial stiffness can be assessed using the central augmentation index adjusted to a heart rate of 75 beats per minute (AIx@75), while vascular aging can be estimated through the vascular aging index (VAI) [23]. The combined use of carotid ultrasound and gold-standard devices such as SphygmoCor^®^ and VaSera^®^ allows for an exhaustive phenotypic characterization of vascular health in patients with LC.

Therefore, the primary aim of this study was to assess the relationship between dietary macronutrient intake and markers of arterial structure, vascular function, and vascular aging in a cohort of patients with LC from the BioICOPER study, with an exploratory analysis of potential sex-related differences. We hypothesized that a more favorable macronutrient profile would be associated with a healthier vascular phenotype, although the cross-sectional design precludes causal inference regarding the role of diet in post-COVID-19 vascular alterations. A better understanding of the role of dietary components in vascular health may help identify key modifiable lifestyle factors for cardiovascular risk reduction in individuals with LC.

## 2. Materials and Methods

### 2.1. Study Design and Participants

A cross-sectional study was conducted in a cohort of 304 adult subjects diagnosed with LC. The study was carried out at the Primary Care Research Unit in Salamanca (APISAL). The results presented here are part of the BioICOPER project, registered in April 2023 at ClinicalTrials.gov (Identifier: NCT05819840). The study protocol has been previously published [24].

**Figure 1:**
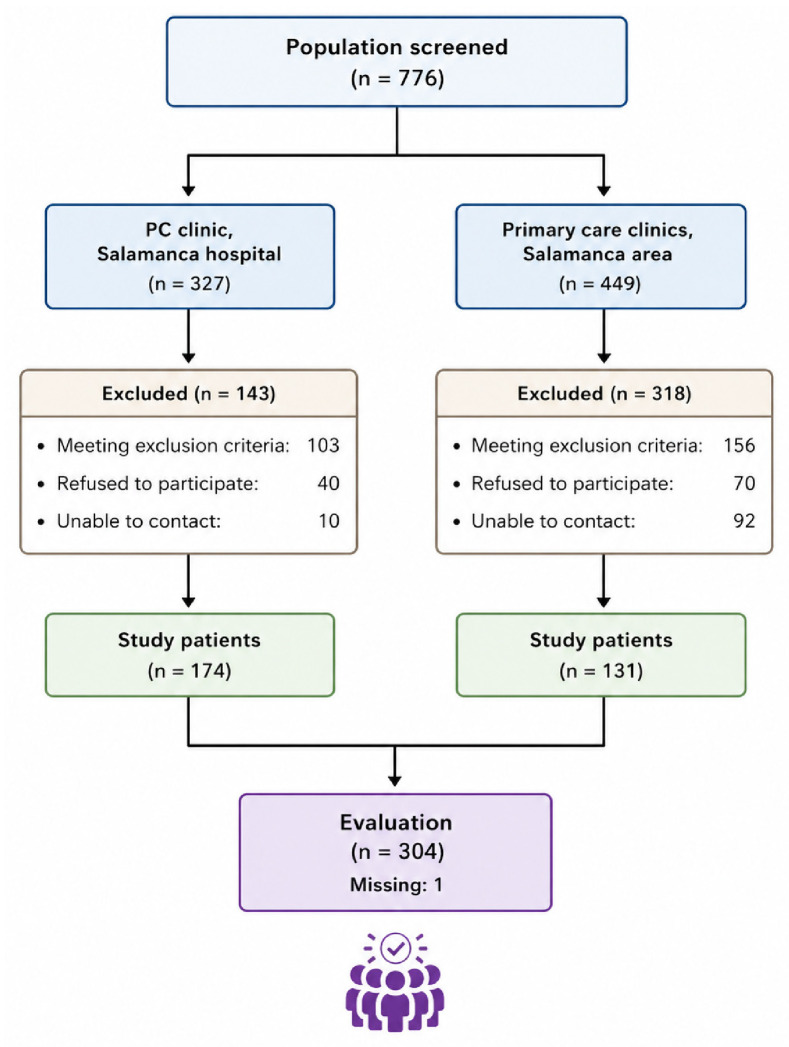
Flowchart of participant selection, including inclusion and exclusion criteria, in the BioICOPER study.

Participants were included through consecutive sampling, between 9 March 2023 and 10 September 2024. Inclusion criteria were: Diagnosis of LC established according to the World Health Organization (WHO) definition [25]. Participants were eligible if they had at least one persistent symptom consistent with LC that lasted at least two months following probable or confirmed COVID-19 caused by SARS-CoV-2 and was not explained by an alternative diagnosis. These symptoms included fatigue or asthenia, dyspnea, cognitive impairment or “brain fog,” sleep disturbances, headache, myalgia or arthralgia, chest pain or palpitations, disturbances in smell or taste, and persistent gastrointestinal symptoms. It was not mandatory to include any specific symptoms, and provision of written informed consent. Exclusion criteria were individuals with terminal illness, those unable to attend the primary care center for assessment, those with a history of cardiovascular disease (ischemic heart disease or cerebrovascular disease), and those with an estimated glomerular filtration rate below 30% (Figure 1).

Sample Size Calculation: The sample size and detectable effect size were estimated using GRANMO software (https://www.datarus.eu/ca/aplications/granmo/, accessed on 12 January 2026). For the multiple linear regression analysis, considering a total sample size of 304 participants, five covariates, an alpha risk of 0.05, a beta risk below 0.20 in a two-sided test, and an expected model R^2^ of 0.347, the study was able to detect a minimum partial correlation of approximately 0.16 between daily energy intake and cfPWV. Based on the observed standard deviations of cfPWV and energy intake, this corresponds to an estimated minimum detectable regression coefficient of approximately 0.06 m/s in cfPWV per 100 kcal/day.

This study followed the Strengthening the Reporting of Observational Studies in Epidemiology (STROBE) guidelines [26], and the complete checklist is provided in Supplementary Table S1.

### 2.2. Variables and Measurement Instruments

To minimize information bias, all analyzed variables and complementary tests were performed by four healthcare professionals who were previously trained before the start of the study, following a standardized protocol. Data were recorded using REDCap 14.5.2 (Research Electronic Data Capture), hosted at the Biomedical Research Institute of Salamanca (IBSAL), for data collection and management. An independent investigator performed quality control [24].

### 2.3. Dietary Intake Assessment

Dietary intake was evaluated using a 7-day food record completed through the EVIDENT mobile application [21]. The EVIDENT application, developed and validated by the Primary Care Research Group of Castilla and León (REDIAPP), is registered under intellectual property number 00/2014/2207. This platform was specifically designed for dietary assessment and enabled participants to document all consumed items over seven consecutive days, including portion sizes and cooking methods. Reported foods and beverages were then categorized into predefined groups within the application. To minimize self-report bias, recall-related errors, under-reporting, and reporting fatigue, participants received standardized instructions to record intake as accurately as possible and preferably close to the time of consumption. The 7-day recording period included both weekdays and weekend days, allowing a more representative estimate of habitual intake. In addition, dietary records were reviewed before nutrient calculation to identify missing information, inconsistencies, or implausible entries. Daily intake of macronutrients was estimated using Spanish food composition tables, including energy (expressed in kilocalories per day), dietary fiber, protein, carbohydrates, and total fat (expressed in grams per day), as well as protein, carbohydrates, and total fat expressed as a percentage of total energy intake [21].

The adequacy of the caloric profile, as well as the intake of fiber, carbohydrates, and fats, was assessed according to the combined criteria of the European Food Safety Authority (EFSA) and the Spanish Society of Community Nutrition (SENC). Specifically, adequate adherence was defined when carbohydrates provided 45–60% of total daily energy intake, total fat accounted for 20–35% [27,28], and protein for 10–15% of total energy intake, in accordance with recommendations for the Spanish population [29]. Finally, dietary fiber intake was classified as adequate when intake was ≥25 g/day, a reference value agreed upon by both institutions [29].

### 2.4. Assessment of Vascular Structure, Function, and Aging

All measurements were performed under baseline conditions, after 10 min of rest in the supine position, in a temperature-controlled room.

#### 2.4.1. Carotid Intima–Media Thickness (cIMT) Measurement

cIMT was measured by two researchers with demonstrated reliability. Interobserver and intraobserver intraclass correlation coefficients (ICCs), calculated in 20 subjects prior to the start of the study, were 0.974 and 0.897, respectively. Measurements were performed using a Sonosite Micromax^®^ ultrasound system (Sonosite Inc., Bothell, WA, USA) equipped with a 5–10 MHz multifrequency high-resolution linear transducer and Sonocal software 1.0 (Washington, DC, USA), which enables automated cIMT measurement [30].

#### 2.4.2. Arterial Stiffness Measurements

Arterial stiffness was assessed by measuring cfPWV, baPWV, CAVI, and AIx@75.

Central arterial stiffness was determined by cfPWV using the SphygmoCor system (AtCor Medical Pty Ltd., West Ryde, Australia), with participants lying supine. Carotid and femoral pressure waveforms were acquired sequentially, and pulse transit time was estimated using the R wave of the electrocardiogram as the reference point. Surface distances from the suprasternal notch to the carotid and femoral recording sites were obtained with a tape measure, and cfPWV was derived following established measurement guidelines [31,32,33].

The AIx@75 was calculated as augmentation pressure divided by pulse pressure and standardized to a heart rate of 75 beats per minute. To standardize measurements and allow comparisons, the AIx@75 [8,9] was calculated according to the following equation [34]:

AIx@75 = AIx + k × (HR − 75)

where AIx represents the augmentation index, HR the heart rate, and k a device-specific correction factor.

baPWV and the CAVI were measured using the VaSera VS-2000 device (Fukuda Denshi Co., Ltd., Tokyo, Japan). Electrodes were attached to both arms and ankles, and measurements were taken while participants remained supine, silent, and still. A microphone sensor was positioned at the second intercostal space to detect heart sounds. CAVI is calculated using an equation derived from the arterial stiffness parameter β, which integrates blood pressure, pulse wave velocity, and blood density to estimate arterial stiffness:

CAVI=a×{[2ρ/(SBP−DBP)]×ln(SBP/DBP)×PWV^2}+b

In this equation, ρ represents blood density, SBP and DBP denote systolic and diastolic blood pressure, respectively, and PWV corresponds to the pulse wave velocity from the heart to the ankle. The logarithmic term, ln(SBP/DBP), accounts for the non-linear relationship between arterial pressure and arterial elasticity. The inclusion of PWV^2^ reflects pulse wave propagation velocity as an indicator of arterial stiffness, whereas the factor 2ρ/(SBP − DBP) adjusts the estimate for pulse pressure. Constants a and b are device-specific calibration parameters provided by the manufacturer. Overall, this formulation allows CAVI to estimate arterial stiffness based on the mechanical properties of the arterial wall, and it is considered less dependent on blood pressure at the time of measurement compared to conventional indices such as pulse wave velocity alone [22,35]. Constants a and b are device-specific calibration parameters. Measurements were considered valid after at least three consecutive stable heartbeats. According to the manufacturer’s instructions, baPWV was calculated using the following equation:

baPWV=(0.5934∗height (cm)+14.4724)/t_ba

baPWV was derived from the ratio between an estimated arterial path length based on body height and the pulse transit time between brachial and ankle sites, reflecting arterial stiffness [8,36,37].

#### 2.4.3. Measurement of the Vascular Aging Index

VAI was calculated as previously described by Wadström et al. [23]. The index was derived by standardizing cIMT and cfPWV to z-scores and summing both components, according to the following formula:

VAI=(cIMT−mean cIMT)/SD cIMT+(cfPWV−mean cfPWV)/SD cfPWV

### 2.5. Sociodemographic Variables, Lifestyle Factors, and Laboratory Analyses

Information on sociodemographic characteristics and lifestyle habits was obtained through standardized questionnaires. Demographic data included age and sex, whereas lifestyle variables comprised smoking status and alcohol intake, assessed using questionnaires adapted from the WHO MONICA study [38]. Adherence to the Mediterranean dietary pattern was assessed with the 14-item Mediterranean Diet Adherence Screener (MEDAS) [39]. Physical activity was measured using a validated digital pedometer (Omron HJ-321-E, Omron Healthcare Co., Ltd., Kyoto, Japan), which records total steps, aerobic steps, distance traveled (km), and calories expended over the previous seven days [40]. Quality of life was assessed using the Short Form Health Survey (SF-36) [41]. After overnight fasting, blood samples were drawn for the determination of plasma glucose concentrations and lipid profile. Blood pressure and heart rate were obtained using a validated automated sphygmomanometer (OMRON M10-IT, Omron Healthcare Co., Ltd., Kyoto, Japan), following European Society of Hypertension recommendations [32].

### 2.6. Statistical Analysis

Statistical analyses were performed using IBM SPSS Statistics version 30.0 (IBM Corp., Armonk, NY, USA). Statistical significance was set at *p* < 0.05. Descriptive analysis: Quantitative variables are presented as mean ± standard deviation (SD), and comparisons between men and women were performed using Student’s *t*-test for independent samples. Qualitative variables are expressed as frequencies and percentages, and comparisons between sexes were conducted using the chi-square test. To evaluate differences in vascular structure, function, and aging according to compliance with dietary recommendations, an analysis of covariance (ANCOVA) was performed. Compliance status (compliant/non-compliant) was included as a fixed factor, and age and sex were included as covariates to control for potential confounding. Results are expressed as estimated marginal means with their standard errors. The linear relationship between continuous variables of vascular structure, function, and aging and macronutrient intake was assessed using Pearson’s correlation coefficient. The magnitude and direction of these associations were visually represented using a heat map. Multivariate analysis: To assess the independent association between vascular structure, function, and aging and macronutrient intake, multiple linear regression models were constructed. Measures of vascular structure (cIMT), arterial stiffness (cfPWV, baPWV, CAVI, and AIx@75), and vascular aging (VAI) were included as dependent variables. Intake of macronutrients (energy, fiber, protein, carbohydrates, and total fat, expressed in grams or as a percentage of total energy intake) were included individually as independent variables. Age, sex, mean blood pressure (MBP) and body mass index (BMI) were included as covariates (Model 1). Model 2 was constructed for those models in which significant associations were identified. In this model, the SF-36 quality of life score and the Mediterranean diet score were included as additional covariates to those already considered in Model 1. Prior to analysis, assumptions of the linear model were verified, and multicollinearity was assessed using the variance inflation factor (VIF), with all values < 2.0. Results are expressed as regression coefficients (β) with their corresponding 95% confidence intervals (95% CI). Finally, to assess potential effect modification by sex, the interaction between sex and dietary variables that showed significant associations was examined in Model 3 by introducing a multiplicative term (sex × each macronutrient variable) into the adjusted multiple linear regression model. It was established a priori that, if this interaction was statistically significant (*p* < 0.05), the analyses would be repeated stratified by sex.

### 2.7. Ethical Considerations

The study protocol was approved by the Ethics Committee for Research with Medicines of the Salamanca Health Area on 27 June 2022 (CEIm Ref. PI 2022 06 1048). The study was conducted in accordance with the principles of the Declaration of Helsinki and the WHO recommendations for good practice in observational research [42]. Participant confidentiality was preserved throughout the study, in compliance with Spanish Organic Law 3/2018, Regulation (EU) 2016/679, and the data protection provisions of 27 April 2016. All participants provided written informed consent before enrolment, after receiving detailed information about the study procedures.

### 2.8. Use of Artificial Intelligence Tools

During manuscript preparation, ChatGPT (based on the GPT-5.5 Thinking model, OpenAI, San Francisco, CA, USA) was employed to assist with English-language editing and with the formatting and assembly of manuscript figures based on author-generated outputs. No generative AI was used to create scientific images, generate data, perform analyses, or alter study results. All figures were checked against the original data and statistical outputs by the authors. All AI-assisted revisions were reviewed and approved by the authors, who assume full responsibility for the final version of the manuscript.

## 3. Results

### 3.1. Characteristics of Participants with LC at Study Inclusion

The characteristics of the study participants, overall and stratified by sex, are presented in Table 1. The proportion of subjects was higher among women (68%). Men were older, reported higher alcohol consumption, and exhibited higher values of systolic blood pressure (SBP), diastolic blood pressure (DBP), fasting plasma glucose (FPG), triglycerides, and BMI. In contrast, women showed higher levels of high-density lipoprotein cholesterol (HDL-C). Regarding vascular structure and function parameters, men displayed higher values across most measures, with the exception of AIx@75, which was higher in women. The mean time elapsed from the diagnosis of acute COVID-19 to study inclusion was 39 ± 10 months.

### 3.2. Table 2 Summarizes Daily Energy and Macronutrient Intake Assessed Using the Evident Tool

No significant differences between sexes were observed for any of the analyzed parameters. Likewise, the proportion of participants meeting the recommended intake for fiber, proteins, carbohydrates, and fats did not differ between men and women.

**Table 2:** Macronutrient Intake Overall and by Sex.

Variable	Overall (n = 304)	Men (n = 97)	Women (n = 207)	*p*
Energy intake (kcal/day)	1800 ± 500	1800 ± 500	1800 ± 500	0.67
Dietary fiber (g/day)	22 ± 7	22 ± 7	22 ± 7	0.81
CD. fiber consumption (n, %)	86 (28%)	28 (29%)	58(28%)	0.49
Protein (g/day)	80 ± 21	82 ± 21	80 ± 20	0.68
Proteins (% of total energy)	19 ± 3	19 ± 3	19 ± 3	0.39
CD. Proteins consumption (n, %)	207 (68%)	63 (65%)	144 (70%)	0.25
Carbohydrates (g/day)	166 ± 61	167 ± 64	165 ± 58	0.25
Carbohydrates (% of total energy)	37 ± 6	37 ± 8	37 ± 9	0.84
CD carbohydrates consumption (n, %)	23 (8%)	9 (9%)	14 (7%)	0.25
Total fat (g/day)	83 ± 24	83 ± 24	83 ± 25	0.29
Total fats (% of total energy)	42 ± 5	42 ± 5	42 ± 6	0.50
CD. total fats consumption (n, %)	18 (6%)	8 (8%)	10 (5%)	0.18

Continuous variables are presented as mean ± standard deviation (SD), and dichotomous variables as number and percentage. CD, consumes adequate.

### 3.3. Estimated Marginal Means of Vascular Structure, Function, and Aging Parameters According to Macronutrient Intake Adherence

**Figure 2:**
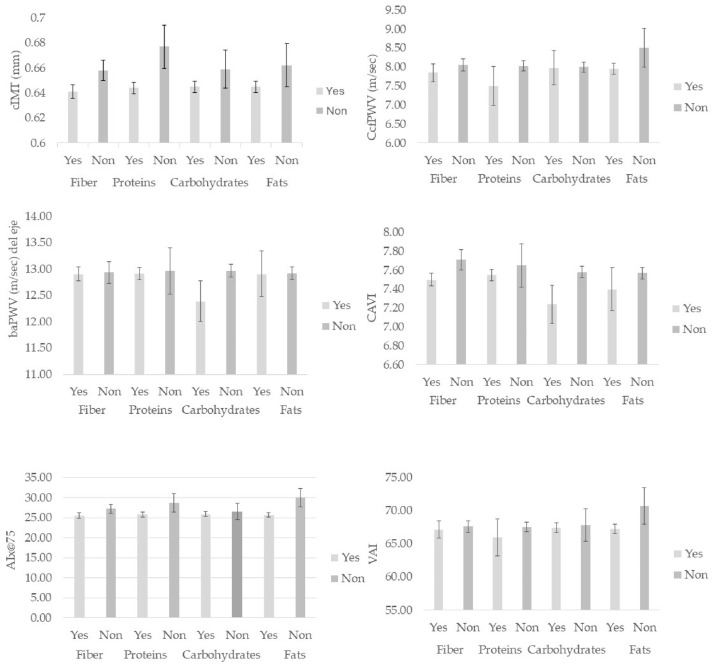
Estimated marginal mean values of cIMT, cfPWV, baPWV, CAVI, AIx@75, and VAI in subjects with and without adequate intake of fiber, proteins, carbohydrates, and fats. cIMT, carotid intima–media thickness; cfPWV, carotid–femoral pulse wave velocity; baPWV, brachial–ankle pulse wave velocity; CAVI, cardio-ankle vascular index; AIx@75, central augmentation index adjusted to a heart rate of 75 bpm; VAI, vascular aging index.

Figure 2 presents the estimated marginal means of carotid intima–media thickness (cIMT), carotid–femoral pulse wave velocity (cfPWV), brachial–ankle pulse wave velocity (baPWV), cardio-ankle vascular index (CAVI), augmentation index adjusted to 75 bpm (AIx@75), and vascular aging index (VAI), adjusted for age and sex, in participants with and without adequate intake of fiber, proteins, carbohydrates, and fats. No statistically significant differences were observed between groups in any of the analyzed parameters (*p* > 0.05 for all comparisons).

### 3.4. Pearson’s Correlation Between Vascular Structure, Function, and Aging Parameters and Macronutrient Intake

**Figure 3:**
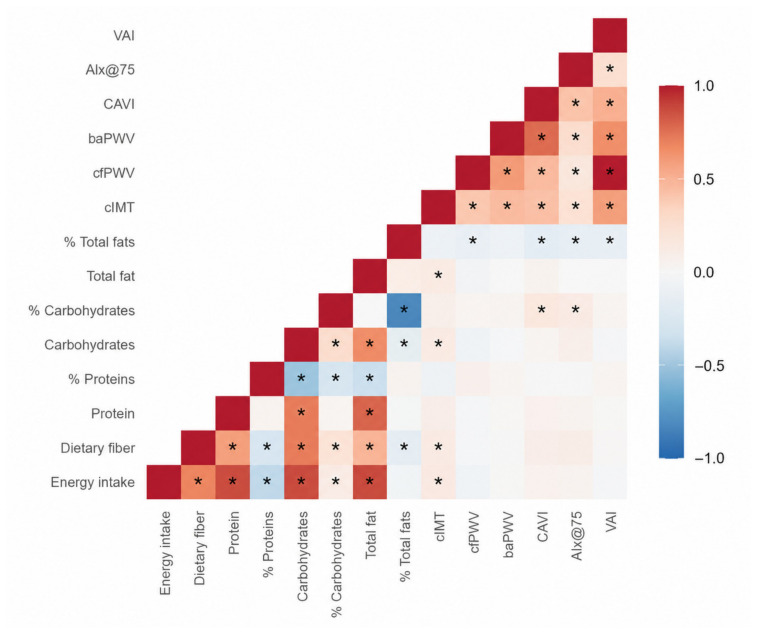
Correlations between parameters of vascular structure, function, and vascular aging and macronutrient intake. cIMT, carotid intima–media thickness; cfPWV, carotid–femoral pulse wave velocity; baPWV, brachial–ankle pulse wave velocity; CAVI, cardio-ankle vascular index; AIx@75, augmentation index adjusted to a heart rate of 75 bpm; VAI, vascular aging index. * *p* < 0.05.

Figure 3 shows the correlations between macronutrient intake parameters and markers of vascular structure, function, and aging. A positive correlation was observed between cIMT and total energy intake (r = 0.13; *p* = 0.02), as well as daily intake of fiber (r = 0.12; *p* = 0.04), carbohydrates (r = 0.12; *p* = 0.04), and fats (r = 0.12; *p* = 0.04). Positive correlations were also found between CAVI (r = 0.14; *p* = 0.01) and AIx@75 (r = 0.13; *p* = 0.02) with the percentage of carbohydrate intake. In contrast, negative correlations were observed between cfPWV (r = −0.12; *p* = 0.04), CAVI (r = −0.15; *p* = 0.01), AIx@75 (r = −0.14; *p* = 0.02), and VAI (r = −0.13; *p* = 0.03) with the percentage of total fat intake.

### 3.5. Multiple Linear Regression Analysis of Vascular Structure, Function, and Aging Parameters in Relation to Macronutrient Intake Assessed by the EVIDENT Tool

The results of the multiple linear regression analysis, using vascular structure, function, and aging measures as dependent variables; macronutrient intake variables as independent variables; and age, sex, MBP, and body mass index (BMI) as adjustment variables, are presented in Table 3. cfPWV remained negatively associated with total energy intake (β = −0.06; 95% CI: −0.11 to −0.01; *p* = 0.02) and carbohydrate intake (β = −0.46; 95% CI: −0.85 to −0.06; *p* = 0.02). No significant associations were observed between macronutrient intake and cIMT, baPWV, CAVI, or VAI. Regarding AIx@75, the percentage of carbohydrate intake relative to total energy was positively associated with AIx@75 (β = 0.79; 95% CI: 0.11 to 1.48; *p* = 0.02), whereas the percentage of total fat intake relative to total energy was inversely associated with AIx@75 (β = −0.29; 95% CI: −0.48 to −0.10; *p* = 0.003).

**Figure 4:**
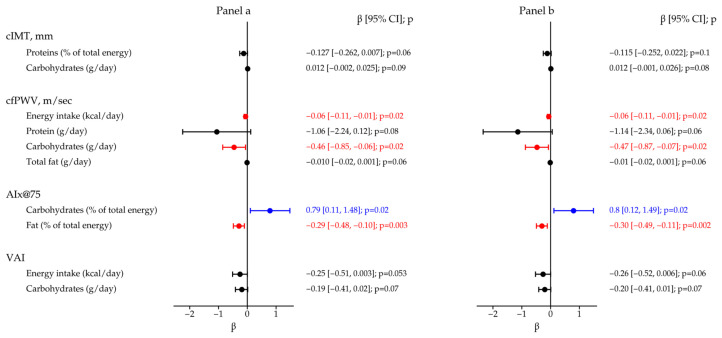
Multiple regression analyses were performed using parameters of vascular structure (cIMT), vascular function (cfPWV and Aix@75), and vascular aging (VAI) as dependent variables. Macronutrient intake and their percentage contribution to total energy intake were included as independent variables. (**Panel a**) shows Model 1, adjusted for age, sex, mean arterial pressure and body mass index. (**Panel b**) shows Model 2, additionally adjusted for SF-36 score and Mediterranean diet score. cIMT: carotid intima–media thickness; cfPWV: carotid–femoral pulse wave velocity; Aix@75: central augmentation index adjusted to 75 beats per minute; VAI: vascular aging index. Red means highlighted in women. Blue means values in men.

Figure 4 shows the associations between dietary macronutrient variables and vascular outcomes that reached statistical significance or showed near-threshold associations, defined as *p* < 0.09 in at least one of the multivariable models. Panel a corresponds to Model 1, adjusted for age, sex, MBP and BMI. Panel b shows Model 2, additionally adjusted for SF-36 score and Mediterranean diet adherence.

For cIMT, percentage of protein intake/total energy showed an inverse association in Model 1 (β = −0.127; 95% CI: −0.262 to 0.007; *p* = 0.06), although this association was attenuated in Model 2 (β = −0.115; 95% CI: −0.252 to 0.022; *p* = 0.1). Carbohydrate intake in g/day showed a positive association near the threshold with cIMT, especially in Model 2 (β = 0.012; 95% CI: −0.001 to 0.026; *p* = 0.08). Regarding cfPWV, energy intake and carbohydrate intake in g/day were inversely associated with cfPWV in both models, remaining statistically significant after additional adjustment in Model 2 (energy intake: β = −0.06; 95% CI: −0.11 to −0.01; *p* = 0.02; carbohydrate intake: β = −0.47; 95% CI: −0.87 to −0.07; *p* = 0.02). Protein intake and total fat intake in g/day also showed inverse near-threshold associations with cfPWV in the fully adjusted model (protein intake: β = −1.14; 95% CI: −2.34 to 0.06; *p* = 0.06; total fat intake: β = −0.01; 95% CI: −0.02 to 0.001; *p* = 0.06).

For AIx@75, carbohydrate intake expressed as percentage of total energy was positively associated with AIx@75 in both models, including Model 2 (β = 0.8; 95% CI: 0.12 to 1.49; *p* = 0.02), whereas fat intake expressed as percentage of total energy showed a consistent inverse association (β = −0.30; 95% CI: −0.49 to −0.11; *p* = 0.002). Finally, VAI showed inverse near-threshold associations with energy intake and carbohydrate intake in g/day in the fully adjusted model (energy intake: β = −0.26; 95% CI: −0.52 to 0.006; *p* = 0.06; carbohydrate intake: β = −0.20; 95% CI: −0.41 to 0.01; *p* = 0.07). These findings suggest that selected macronutrient intake variables are associated with vascular structure, central arterial stiffness, wave reflection, and vascular aging markers, with several associations persisting after adjustment for health-related quality of life and adherence to the Mediterranean diet.

Finally, in those measures with significance in model 2, we have analyzed the interaction terms, between sex and dietary intake variables were included in the models. No significant sex differences were observed for the associations between cfPWV and energy intake (*p* for interaction = 0.678), cfPWV and carbohydrate intake (*p* for interaction = 0.497), or Aix@75 and % carbohydrate intake/energy intake (*p* for interaction = 0.453), Aix@75 and % Fat intake/energy intake (*p* for interaction = 0.914).

## 4. Discussion

### 4.1. Main Findings

The present study provides novel insights into the relationship between dietary macronutrient intake and vascular health in patients with LC. To the best of our knowledge, this is one of the few studies integrating detailed dietary assessment with a comprehensive multimodal evaluation of vascular structure, arterial stiffness, wave reflection, and vascular aging in this specific population.

The main findings show that selected dietary macronutrient variables were independently associated with markers of central vascular function in patients with LC. In the fully adjusted model, total energy intake and carbohydrate intake in g/day were inversely associated with central arterial stiffness, as assessed by cfPWV. Carbohydrate intake expressed as percentage of total energy was positively associated with AIx@75, whereas fat intake expressed as percentage of total energy showed an inverse association with AIx@75. By contrast, the associations with cIMT and VAI were only near-threshold, no significant differences were observed according to compliance with macronutrient intake recommendations, and no significant associations were found for CAVI in the fully adjusted model. Taken together, these findings suggest that the relationship between dietary macronutrient intake and vascular health in LC is complex and depends on the vascular parameter evaluated. Absolute macronutrient intake appeared to be more closely related to central arterial stiffness, whereas the relative distribution of macronutrients was associated with wave reflection. This parameter-dependent pattern is consistent with the broader concept that macronutrient-health relationships may be non-linear and interactive rather than attributable to isolated nutrients alone, as also suggested by multi-nutrient analyses from NHANES [43].

### 4.2. Absolute Macronutrient Intake, Vascular Structure, and Central Arterial Stiffness

The near-threshold inverse association between protein intake expressed as percentage of total energy and cIMT observed in Model 1 should be interpreted with caution, because it was attenuated after additional adjustment for health-related quality of life and adherence to the Mediterranean diet. Nevertheless, this finding is partially consistent with previous evidence in non-COVID populations suggesting that higher protein intake, particularly from plant-based or high-quality protein sources, may be associated with a more favorable cardiovascular risk profile [44,45,46]. In our study, protein intake in g/day also showed an inverse near-threshold association with cfPWV in the fully adjusted model, suggesting a possible relationship with central arterial stiffness rather than a definitive effect on vascular structure.

However, the evidence specifically linking protein intake to cIMT remains limited and inconsistent, and no causal interpretation can be drawn from our cross-sectional design. Moreover, the vascular effects of protein intake may depend not only on the total amount consumed, but also on protein quality, food source, amino acid profile, and the inflammatory or metabolic context associated with different dietary patterns. Previous studies have also reported inverse associations between plant-based protein intake and all-cause and cardiovascular mortality [47], supporting the need to further explore the role of protein source in vascular health among patients with LC.

In the fully adjusted model, total energy intake and carbohydrate intake in g/day were inversely associated with cfPWV, while total fat intake in g/day showed an inverse near-threshold association. These associations were observed for cfPWV, a clinically relevant marker of central arterial stiffness and cardiovascular risk [13]. Although the direction of these associations may appear counterintuitive, several considerations should be taken into account. First, in individuals with LC, lower energy and macronutrient intake may reflect worse clinical status, reduced appetite, fatigue, functional limitation, or disease-related nutritional compromise rather than a healthier dietary pattern. Therefore, reduced intake could act as a surrogate marker of disease burden. This interpretation is supported by evidence suggesting that inadequate nutritional status may exacerbate systemic inflammation, oxidative stress, and endothelial dysfunction [1,48]. Second, our analysis focused primarily on the amount of macronutrients consumed rather than their quality or food sources. Consequently, potentially beneficial and harmful dietary patterns may be grouped within the same macronutrient category. In this regard, nutrient quality, such as the distinction between refined and complex carbohydrates or between saturated and unsaturated fats, may be a key determinant of vascular health beyond the absolute amount consumed [49,50]. Therefore, the inverse associations between absolute energy or carbohydrate intake and cfPWV should not be interpreted as evidence that higher intake per se is more beneficial, but rather as an indication that nutritional status and dietary adequacy may be relevant in the vascular phenotype of patients with LC.

### 4.3. Macronutrient Distribution, CAVI, and Wave Reflection

No significant association between carbohydrate intake and CAVI was observed in the fully adjusted model. This suggests that, after accounting for health-related quality of life and adherence to the Mediterranean diet, the relationship between macronutrient intake and arterial stiffness as assessed by CAVI was not robust in this cohort. This null finding is relevant because CAVI reflects a blood pressure-independent estimate of arterial stiffness and may be particularly influenced by global cardiometabolic phenotypes, including obesity and metabolic syndrome [51]. In contrast, macronutrient distribution was significantly associated with AIx@75, a marker related to wave reflection and arterial tone. Specifically, carbohydrate intake expressed as percentage of total energy was positively associated with AIx@75, whereas fat intake expressed as percentage of total energy showed an inverse association. These findings suggest that the relative contribution of macronutrients to total energy intake may be more closely related to wave reflection than to CAVI-derived arterial stiffness in patients with LC.

### 4.4. Mechanistic Perspective

From a mechanistic perspective, the observed associations between macronutrient distribution and AIx@75 can be interpreted within the broader framework of endothelial dysfunction described in patients with LC. COVID-19 caused by SARS-CoV-2 has been associated with persistent endothelial activation, chronic inflammation, oxidative stress, impaired nitric oxide bioavailability, and microvascular damage [52,53,54]. In this context, a higher relative contribution of carbohydrates could reflect, in some individuals, a poorer quality of carbohydrates consumed, a higher glycemic load or a lower overall quality of the diet, factors that could affect endothelial nitric oxide-dependent vasodilation and contribute to alterations in vascular tone and wave reflection [51]. Conversely, the inverse association between fat intake as a percentage of total energy and AIx@75 should be interpreted with caution, as our analysis did not differentiate between saturated, monounsaturated, and polyunsaturated fatty acids, which can exert divergent vascular effects [49,50]. In addition, other nutritional factors not evaluated in the present study, such as amino acids involved in the arginine–nitric oxide pathway and vitamin D status, could also modulate endothelial function. Vitamin D is particularly relevant, as the vitamin D receptor is expressed in endothelial cells and has been linked to the regulation of inflammation, immunometabolic function, oxidative stress, and endothelial nitric oxide synthase activity [52,53]. Therefore, these findings do not support a direct adverse effect of carbohydrate intake on all arterial stiffness indices, but suggest a possible relationship between macronutrient distribution, endothelial tone, and wave reflection. Since vitamin D concentrations, arginine/citrulline metabolism, nitric oxide-related biomarkers, oxidative stress markers, and detailed carbohydrate and fatty acid quality profiles were not assessed, these mechanistic interpretations should be considered exploratory. Future studies integrating dietary assessment with biochemical biomarkers will be needed to clarify the pathways linking nutrition, endothelial function, and arterial stiffness in people with LC.

### 4.5. Sex-Related Differences in Vascular Findings

The sex-related differences observed in the present study deserve further consideration, especially in the context of vascular alterations following COVID-19. In our cohort, men showed higher values for most vascular structure and stiffness-related parameters, including cIMT, cfPWV, baPWV, CAVI, and VAI, whereas AIx@75 was higher in women. These differences indicate that the vascular phenotype of LC may not be homogeneous between sexes and may help contextualize the associations observed between dietary intake and vascular outcomes. Sex can influence vascular function through differences in endothelial regulation, inflammatory response, hormonal status, body composition, fat distribution, and cardiometabolic risk profile. Recent evidence supports the relevance of this issue. Durieux et al. [7] reported that sex modified the effect of COVID-19 on arterial elasticity, with women with post-acute sequelae of COVID-19 showing the highest augmentation index, suggesting poorer arterial elasticity. These findings are consistent with the hypothesis that vascular deterioration following COVID-19 may not be homogeneous between sexes. Although the vascular profile differed between men and women, interaction analyses did not support a statistically significant modifying effect of sex on the associations between dietary macronutrient intake and vascular parameters. Therefore, sex-stratified findings should be interpreted cautiously and considered exploratory, particularly given the smaller number of men in the cohort. However, these results should be interpreted with caution due to the cross-sectional design and the limited statistical power of some subgroup analyses. Therefore, our findings should not be interpreted as definitive evidence that sex modifies the association between macronutrient intake and vascular damage, but rather as hypothesis-generating evidence that sex may be an important contextual factor in the vascular assessment of patients with LC. Future longitudinal studies with larger samples are needed to clarify whether sex acts as a true effect modifier in the association between nutritional status and arterial stiffness in people with LC.

### 4.6. Clinical and Research Implications

From a clinical perspective, these findings highlight that dietary intake may differentially relate to vascular structure and function depending on the parameter evaluated. The most consistent associations were observed for central vascular function and wave reflection, whereas findings for vascular structure, peripheral stiffness, CAVI, and VAI were weaker or absent. This suggests that dietary macronutrient intake should be interpreted within a broader vascular and cardiometabolic context rather than as a single isolated determinant of vascular damage. The stability of several associations after progressive adjustment supports the robustness of the observed findings, although causality cannot be established due to the cross-sectional design of the study. Moreover, the overall magnitude of the associations was small. Therefore, macronutrient intake should not be considered a standalone therapeutic target, but rather a modest and potentially modifiable factor that should be integrated into a broader dietary and lifestyle strategy aimed at improving cardiovascular health in patients with LC. Future research should move beyond total macronutrient quantity and incorporate more detailed measures of diet quality, food sources, carbohydrate type, glycaemic load, fatty acid profile, and dietary patterns. Longitudinal and interventional studies are also needed to determine whether changes in dietary intake are associated with improvement or stabilization of arterial stiffness, wave reflection, and vascular aging over time in individuals with LC.

The apparently different associations observed for carbohydrate intake in g/day and carbohydrate intake expressed as percentage of total energy should be interpreted in light of the fact that these variables represent different nutritional constructs. Absolute carbohydrate intake may partly reflect overall energy intake and nutritional status, whereas carbohydrate intake as a percentage of total energy reflects macronutrient distribution and potential substitution effects. Moreover, cfPWV and AIx@75 assess different components of vascular function: cfPWV primarily reflects central arterial stiffness, while AIx@75 is influenced by wave reflection, arterial tone and peripheral vascular properties.

### 4.7. Limitations and Strengths of the Study

This study has several limitations that should be taken into account when interpreting the results. First, its observational and cross-sectional design prevents causal inference. Therefore, the observed associations between macronutrient intake and vascular parameters should not be interpreted as evidence of a causal effect. Reverse causality cannot be excluded, as individuals with greater vascular impairment, higher symptom burden, fatigue, functional limitations, or poorer general health may have modified their eating habits. Residual confounding cannot be excluded either. Although the models were adjusted for relevant clinical and lifestyle variables, other unmeasured factors may have influenced both dietary intake and vascular outcomes. Therefore, residual confounding may have affected the magnitude or direction of some associations. Second, several dietary variables were examined in relation to multiple vascular outcomes. Although the analyses were based on predefined vascular parameters and biologically plausible dietary exposures, this approach increases the possibility of type I error, especially for associations with *p*-values close to 0.05 and modest effect sizes. Therefore, these findings should be interpreted with caution and considered hypothesis-generating. They require confirmation in future studies with larger sample sizes, prespecified primary endpoints, correction strategies for multiple comparisons when appropriate, and longitudinal or interventional designs. Third, dietary intake was assessed using a 7-day food record. Although this method provides detailed dietary information, it is subject to self-report bias and may not fully capture usual dietary patterns over the long term. In particular, under-reporting of total energy intake or specific foods, recall-related errors when foods were not recorded immediately after consumption, and reporting fatigue over the 7-day period cannot be excluded. Although these potential biases were minimized through the use of a structured dietary tool, standardized instructions, and review of dietary records to identify missing information, inconsistencies, or implausible entries, measurement error may still have affected dietary estimates. In addition, this analysis focused primarily on broad categories of macronutrients and their percentage contribution to total energy intake. Therefore, the findings should be interpreted as associations with global macronutrient intake variables rather than with diet quality, specific dietary patterns, or individual cardioprotective foods and nutrients. Fourth, the nutritional database used in the EVIDENT application did not allow detailed differentiation of relevant nutrient subtypes, such as simple versus complex carbohydrates, glycemic index or glycemic load, soluble versus insoluble fiber, or saturated, monounsaturated, and polyunsaturated fatty acids. This is relevant because these components may exert different effects on endothelial function, lipid metabolism, inflammation, insulin resistance, arterial tone, and arterial stiffness. Similarly, the interpretation of protein intake is limited because the vasoactive properties of dietary proteins may depend on their food source, amino acid composition, and the presence of bioactive peptides. Fifth, several aspects of overall diet quality and specific food groups were not comprehensively assessed. Although adherence to the Mediterranean diet was taken into account in the adjusted models, the study was not designed to assess in detail the vascular effects of fruit and vegetable intake, dietary patterns, milk and dairy products, cocoa or other flavanol-rich foods, or the overall quality of carbohydrate, fat, and protein sources. This is important because fruits and vegetables, Mediterranean dietary patterns, dairy products, and cocoa-derived flavanols and procyanidins may influence vascular health through effects on blood pressure, endothelial nitric oxide bioavailability, oxidative stress, inflammation, lipid profile, insulin resistance, and platelet function. Sodium and potassium intake were also not specifically analyzed, despite their close relationship with blood pressure regulation, endothelial function, and arterial stiffness. Sixth, biochemical biomarkers related to endothelial function, inflammation, oxidative stress, and nutritional status were not available. In particular, serum vitamin D concentrations, markers of the arginine–nitric oxide pathway, citrulline, nitric oxide-related biomarkers, oxidative stress markers, and inflammatory mediators such as *C*-reactive protein, IL-6, or TNF-α were not evaluated. Therefore, although the discussion includes plausible pathophysiological mechanisms linking diet, endothelial dysfunction, nitric oxide bioavailability, inflammation, oxidative stress, and vascular stiffness, the present study cannot directly confirm the biochemical pathways underlying the observed associations. In addition, some covariates included in the fully adjusted models, such as blood pressure, may lie on the causal pathway between diet and vascular damage and could therefore act as mediators. As a result, the magnitude of some associations may have been underestimated. Finally, although the sample size provided sufficient statistical power to detect moderate associations in the overall study population, it may have been insufficient to identify small effect sizes, especially after adjustment for multiple covariates and in subgroup or interaction analyses. This limitation is particularly relevant for sex-related analyses, because men represented only approximately one third of the study population. In the revised analyses, sex interaction terms were assessed only for diet–vascular associations that remained significant in the fully adjusted models, and no significant interactions were observed. However, the absence of statistically significant interaction terms should be interpreted with caution and should not be considered definitive evidence that these associations are identical in men and women. Therefore, a type II error cannot be excluded, particularly for sex-specific or subgroup analyses. Future studies with larger samples and a more balanced sex distribution are needed to clarify whether sex modifies the relationship between macronutrient intake and vascular aging in Long COVID.

Among the strengths of this study, we highlight: to our knowledge, one of the first to analyze the association between macronutrient intake, assessed using a validated dietary tool, and multiple parameters of vascular structure, function, and vascular aging in patients with LC. The use of a comprehensive vascular assessment, including cIMT, cfPWV, baPWV, CAVI, AIx@75, and VAI, allowed a broad characterization of the vascular tree. In addition, the use of multivariable models adjusted for relevant clinical and lifestyle factors, together with the consideration of sex-related differences, strengthens the interpretation and clinical relevance of the findings.

## 5. Conclusions

These findings suggest that dietary macronutrient intake may be associated with central arterial stiffness and wave reflection in patients with LC. However, given the cross-sectional design, causality and directionality cannot be established, and the results should be considered hypothesis-generating. Longitudinal and interventional studies are needed to determine whether dietary modification can influence vascular outcomes in this population.

## Figures and Tables

**Table 1:** Characteristics of the study population overall and by sex.

Variable	Overall (n = 304)	Men (n = 97)	Women (n = 207)	*p*
Age, years (mean ± SD)	53 ± 12	56 ± 12	51 ± 12	0.001
Time of evolution, months (mean ± SD)	39 ± 10	39 ± 10	39 ± 9	0.99
SF-36 HTS mean ± SD	50 ± 25	52 ± 25	50 ± 26	0.49
Alcohol, g/week (mean ± SD)	29 ± 53	60 ± 76	15 ± 27	<0.001
No alcohol consumption (n, %)	163 (54)	36 (37)	127 (61)	<0.001
MD score (mean ± SD)	7.8 ± 2.3	7.7 ± 2.2	7.8 ± 2.4	0.44
MD adhesion (n, %)	123 (41)	38 (39)	85 (41)	0.43
Number of steps per day (mean ± SD)	7800 ± 4600	7100 ± 4700	8200 ± 4600	0.03
Current smoker, (n, %)	17 (6)	8 (8)	9 (5)	0.07
No. of cigarettes, (day)	16 ± 11	20 ± 11	13 ± 10	<0.001
SBP, mmHg (mean ± SD)	120 ± 17	129 ± 14	116 ± 16	<0.001
DBP, mmHg (mean ± SD)	77 ± 11	82 ± 11	74 ± 10	<0.001
MBP, mmHg (mean ± SD)	91 ± 12	98 ± 11	88 ± 12	<0.001
Hypertension, (n, %)	109 (35)	52 (54)	57 (27)	<0.001
Total cholesterol, mg/dL (mean ± SD)	187 ± 34	182 ± 33	190 ± 35	0.03
LDL-c, mg/dL (mean ± SD)	113 ± 32	114 ± 32	113 ± 32	0.42
HDL-c, mg/dL (mean ± SD)	57 ± 14	49 ± 11	61 ± 13	<0.001
Triglycerides, mg/dL (mean ± SD)	102 ± 51	117 ± 54	95 ± 48	<0.001
Dyslipidemia, (n, %)	201 (66)	71 (73)	130 (63)	0.053
FPG, mg/dL (mean ± SD)	88 ± 18	94 ± 20	85 ± 16	<0.001
Diabetes mellitus, (n, %)	37 (12.2)	22 (22.7)	15 (7.3)	<0.001
Weight, kg (mean ± SD)	76 ± 17	88 ± 15	70 ± 15	<0.001
Height, cm (mean ± SD)	164.5 ± 8.7	172.5 ± 7.4	160.8 ± 6.5	<0.001
BMI, kg/m^2^ (mean ± SD)	28.0 ± 5.6	29.6 ± 4.6	27.2 ± 5.8	<0.001
Obesity, (n, %)	99 (33)	44 (45)	55 (26)	<0.001
cIMT, mm	0.64 ± 0.09	0.68 ± 0.12	0.62 ± 0.07	<0.001
cfPWV, m/s	7.7 ± 2.4	8.9 ± 3.0	7.1 ± 1.8	<0.001
baPWV, m/seg	12.8 ± 2.4	13.6 ± 2.4	12.4 ± 2.3	<0.001
CAVI	7.5 ± 1.3	7.9 ± 1.4	7.3 ± 1.2	<0.001
AIx@75	28 ± 12	23 ± 11	30 ± 12	<0.001
VAI	66 ± 14	73 ± 17	62 ± 11	<0.001

Continuous variables are presented as mean ± standard deviation, between-sex comparisons were performed using Student’s *t* test. Categorical variables are shown as n (%) and were compared with χ^2^ or Fisher’s exact test, as appropriate. A *p*-value < 0.05 was considered statistically significant. Abbreviations: SD, Standard Deviation; SF-36 HTS, Short Form-36 Health Transition Score; MD, Mediterranean Diet; MET, Metabolic Equivalent; SBP, Systolic Blood Pressure; DBP, Diastolic Blood Pressure; MBP, Mean Blood Pressure; LDL, Low-Density Lipoprotein; HDL, High-Density Lipoprotein; FPG, Fasting Plasma Glucose; BMI, Body Mass Index; cIMT: Carotid intima–media thickness; cfPWV, Carotid–Femoral Pulse Wave Velocity; baPWV, Brachial–Ankle Pulse Wave Velocity; CAVI: Cardio-Ankle Vascular Index; AIx@75, Central Augmentation Index Adjusted to 75 beats per minute; VAI, Vascular Aging Index.

**Table 3:** Associations between parameters of vascular structure, function, and vascular aging and macronutrient intake: multiple linear regression analysis.

cIMT, mm	β	95% (CI)	*p*
Energy intake (kcal/day)	0.001	−0.001 to 0.003	0.14
Fiber (g/day)	0.001	−0.001 to 0.002	0.11
Protein (g/day)	0.008	−0.031 to 0.048	0.68
Proteins (% of total energy)	−0.127	−0.262 to 0.007	0.06
Carbohydrates (g/day)	0.012	−0.002 to 0.025	0.09
Carbohydrates (% of total energy)	0.326	−0.176 to 0.828	0.20
Total fat (g/day)	0.0003	−0.0001 to 0.0006	0.11
Fat (% of total energy)	0.016	−0.124 to 0.157	0.82
cfPWV, m/s			
Energy intake (kcal/day)	−0.06	−0.11 to −0.01	0.02
Fiber (g/day)	−0.02	−0.05 to 0.01	0.21
Protein (g/day)	−1.06	−2.24 to 0.12	0.08
Proteins (% of total energy)	0.31	−0.09 to 0.70	0.13
Carbohydrates (g/day)	−0.46	−0.85 to −0.06	0.02
Carbohydrates (% of total energy)	−0.94	−1.44 to 0.56	0.22
Total fat (g/day)	−0.010	−0.02 to 0.000	0.06
Fat (% of total energy)	0.22	−0.20 to 0.64	0.31
baPWV, m/seg			
Energy intake (kcal/day)	−0.03	−0.07 to 0.006	0.10
Fiber (g/day)	−0.01	−0.04 to 0.01	0.28
Protein (g/day)	−0.71	−1.66 to 0.24	0.14
Proteins (% of total energy)	0.12	−0.20 to 0.44	0.47
Carbohydrates (g/day)	−0.24	−0.56 to 0.08	0.14
Carbohydrates (% of total energy)	−0.53	−1.74 to 0.69	0.40
Total fat (g/day)	−0.006	−0.01 to 0.002	0.12
Fat (% of total energy)	0.11	−0.23 to 0.45	0.52
CAVI			
Energy intake (kcal/day)	−0.02	−0.28 to 0.24	0.90
Fiber (g/day)	0.04	−0.14 to 0.21	0.70
Protein (g/day)	−0.11	−0.74 to 0.52	0.72
Proteins (% of total energy)	0.02	−0.21 to 0.21	0.98
Carbohydrates (g/day)	−0.04	−1.17 to 1.08	0.97
Carbohydrates (% of total energy)	−0.18	−0.98 to 0.63	0.67
Total fat (g/day)	−0.007	−0.06 to 0.05	0.80
Fat (% of total energy)	0.54	−1.71 to 2.78	0.64
AIx@75			
Energy intake (kcal/day)	0.01	−0.21 to 0.24	0.91
Fiber (g/day)	0.08	−0.07 to 0.23	0.32
Protein (g/day)	0.75	−4.67 to 6.17	0.79
Proteins (% of total energy)	0.68	−1.14 to 2.50	0.46
Carbohydrates (g/day)	1.17	−0.64 to 2.98	0.20
Carbohydrates (% of total energy)	0.79	0.11 to 1.48	0.02
Total fat (g/day)	−0.03	−0.07 to 0.02	0.28
Fat (% of total energy)	−0.29	−0.48 to −0.10	0.003
VAI			
Energy intake (kcal/day)	−0.25	−0.51 to 0.003	0.053
Fiber (g/day)	−0.07	−0.25 to 0.10	0.41
Protein (g/day)	−0.51	−1.13 to 0.11	0.11
Proteins (% of total energy)	1.13	−0.99 to 3.25	0.30
Carbohydrates (g/day)	−0.19	−0.41 to 0.02	0.07
Carbohydrates (% of total energy)	−0.37	−1.16 to 0.42	0.36
Total fat (g/day)	−0.04	−0.09 to 0.01	0.13
Fat (% of total energy)	0.12	−0.10 to 0.34	0.29

Multiple regression analyses were performed using parameters of vascular structure (cIMT), vascular function (cfPWV, baPWV, CAVI, and AIx@75), and vascular aging (VAI) as dependent variables. Macronutrient intake and their percentage contribution to total energy intake were included as independent variables. All models were adjusted for age, sex, mean arterial pressure and body mass index. cIMT: carotid intima–media thickness; cfPWV: carotid–femoral pulse wave velocity; baPWV: brachial–ankle pulse wave velocity; CAVI: cardio-ankle vascular index; AIx@75: central augmentation index adjusted to 75 beats per minute; VAI: vascular aging index.

## Data Availability

The data supporting the findings of this study are available on ZENODO at: https://doi.org/10.5281/zenodo.14282873.

## Supplementary Materials

The following supporting information can be downloaded at https://www.mdpi.com/article/10.3390/nu18122028/s1, Table S1: STROBE checklist.

## Abbreviations

The following abbreviations are used in this manuscript:

LC	Long COVID
cIMT	carotid intima–media thickness
cfPWV	carotid–femoral pulse wave velocity
baPWV	brachial–ankle pulse wave velocity
CAVI	cardio-ankle vascular index
AIx@75	central augmentation index adjusted to 75 bpm
VAI	vascular aging index
APISAL	Salamanca Primary Care Research Unit
WHO	World Health Organization
STROBE	Observational Studies in Epidemiology
REDCap	Research Electronic Data Capture
IBSAL	Biomedical Research Institute of Salamanca
PP	pulse pressure
AIx	represents the augmentation index
HR	heart rate
SBP	systolic blood pressure,
DBP	diastolic blood pressure
MBP	Mean blood pressure
PWV	pulse wave velocity
FPG	Fasting plasma glucose
MEDAS	Mediterranean Diet Adherence Screener
HDL	High Density Lipoprotein
SF-36	Short Form Health Survey
BMI	Body mass index
VIF	Variance Inflation Factor
CI	Confidence interval

## References

[1] Davis H.E., McCorkell L., Vogel J.M., Topol E.J. (2023). Long COVID: Major findings, mechanisms and recommendations. Nat. Rev. Microbiol..

[2] Al-Aly Z., Topol E. (2024). Solving the puzzle of Long Covid. Science.

[3] Gelhorn H.L., Ghafoori P., Cutts K., Birch H., Savva Y., Satram S., Lloyd E., Chen W.H. (2024). Characterizing health-related quality of life and identifying disease predictors among patients suspected of having long COVID: An analysis of COMET-ICE clinical trial data. Front. Public Health.

[4] Stojanovic M., Djuric M., Nenadic I., Bojic S., Andrijevic A., Popovic A., Pesic S. (2025). Vascular Complications of Long COVID—From Endothelial Dysfunction to Systemic Thrombosis: A Systematic Review. Int. J. Mol. Sci..

[5] Cervia-Hasler C., Brüningk S.C., Hoch T., Fan B., Muzio G., Thompson R.C., Ceglarek L., Meledin R., Westermann P., Emmenegger M. (2024). Persistent complement dysregulation with signs of thromboinflammation in active Long Covid. Science.

[6] Theresa C., Katebe B., Shibao C.A., Kirabo A. (2024). Arterial stiffness in adults with Long COVID in sub-Saharan Africa. Physiol. Rep..

[7] Durieux J.C., Zisis S.N., Mouchati C., Labbato D., Abboud M., McComsey G.A. (2024). Sex Modifies the Effect of COVID-19 on Arterial Elasticity. Viruses.

[8] Ohkuma T., Ninomiya T., Tomiyama H., Kario K., Hoshide S., Kita Y., Inoguchi T., Maeda Y., Kohara K., Tabara Y. (2017). Brachial-Ankle Pulse Wave Velocity and the Risk Prediction of Cardiovascular Disease: An Individual Participant Data Meta-Analysis. Hypertension.

[9] Cheong S.S., Samah N., Che Roos N.A., Ugusman A., Mohamad M.S.F., Beh B.C., Zainal I.A., Aminuddin A. (2024). Prognostic value of pulse wave velocity for cardiovascular disease risk stratification in diabetic patients: A systematic review and meta-analysis. J. Diabetes Complicat..

[10] Li J., Gao F., Cao F., Lv S., Hou Y., Guo W., Zhang C., Liu A. (2025). Association of estimated pulse wave velocity with cardiovascular disease outcomes and all-cause death-a systematic review and meta-analysis. Front. Cardiovasc. Med..

[11] Prelević V., Blagus L., Bošnjak V., Radunović D., Marinović Glavić M., Premužić V., Kos J., Pećin I., Željković Vrkić T., Domislović M. (2024). Estimated Pulse Wave Velocity and All-Cause and Cardiovascular Mortality in the General Population. J. Clin. Med..

[12] Vieceli T., Brambilla B., Pereira R.Q., Dellamea B.S., Stein A.T., Grezzana G.B. (2021). Prediction of all-cause and cardiovascular mortality using central hemodynamic indices among elderly people: Systematic review and meta-analysis. Sao Paulo Med. J..

[13] Vlachopoulos C., Aznaouridis K., Stefanadis C. (2010). Prediction of cardiovascular events and all-cause mortality with arterial stiffness: A systematic review and meta-analysis. J. Am. Coll. Cardiol..

[14] Stanek A., Grygiel-Górniak B., Brożyna-Tkaczyk K., Myśliński W., Cholewka A., Zolghadri S. (2023). The Influence of Dietary Interventions on Arterial Stiffness in Overweight and Obese Subjects. Nutrients.

[15] Campbell M.S., Fleenor B.S. (2018). Whole grain consumption is negatively correlated with obesity-associated aortic stiffness: A hypothesis. Nutrition.

[16] Kelly R.K., Tong T.Y.N., Watling C.Z., Reynolds A., Piernas C., Schmidt J.A., Papier K., Carter J.L., Key T.J., Perez-Cornago A. (2023). Associations between types and sources of dietary carbohydrates and cardiovascular disease risk: A prospective cohort study of UK Biobank participants. BMC Med..

[17] Chiang W.L., Azlan A., Yusof B.M. (2023). Sugar Consumption Pattern among Cardiometabolic Risk Individuals: A Scoping Review. Curr. Diabetes Rev..

[18] Reynolds A., Mann J., Cummings J., Winter N., Mete E., Te Morenga L. (2019). Carbohydrate quality and human health: A series of systematic reviews and meta-analyses. Lancet.

[19] Barber T.M., Kabisch S., Pfeiffer A.F.H., Weickert M.O. (2020). The Health Benefits of Dietary Fibre. Nutrients.

[20] Ma Y., Zheng Z., Zhuang L., Wang H., Li A., Chen L., Liu L. (2024). Dietary Macronutrient Intake and Cardiovascular Disease Risk and Mortality: A Systematic Review and Dose-Response Meta-Analysis of Prospective Cohort Studies. Nutrients.

[21] Recio-Rodriguez J.I., Rodriguez-Martin C., Gonzalez-Sanchez J., Rodriguez-Sanchez E., Martin-Borras C., Martínez-Vizcaino V., Arietaleanizbeaskoa M.S., Magdalena-Gonzalez O., Fernandez-Alonso C., Maderuelo-Fernandez J.A. (2019). EVIDENT Smartphone App, a New Method for the Dietary Record: Comparison with a Food Frequency Questionnaire. JMIR mHealth uHealth.

[22] Shirai K., Hiruta N., Song M., Kurosu T., Suzuki J., Tomaru T., Miyashita Y., Saiki A., Takahashi M., Suzuki K. (2011). Cardio-ankle vascular index (CAVI) as a novel indicator of arterial stiffness: Theory, evidence and perspectives. J. Atheroscler. Thromb..

[23] Nilsson Wadström B., Fatehali A.H., Engström G., Nilsson P.M. (2019). A Vascular Aging Index as Independent Predictor of Cardiovascular Events and Total Mortality in an Elderly Urban Population. Angiology.

[24] Gómez-Sánchez L., Tamayo-Morales O., Suárez-Moreno N., Bermejo-Martín J.F., Domínguez-Martín A., Martín-Oterino J.A., Martín-González J.I., González-Calle D., García-García Á., Lugones-Sánchez C. (2023). Relationship between the structure, function and endothelial damage, and vascular ageing and the biopsychological situation in adults diagnosed with persistent COVID (BioICOPER study). A research protocol of a cross-sectional study. Front. Physiol..

[25] Soriano J.B., Murthy S., Marshall J.C., Relan P., Diaz J.V. (2022). A clinical case definition of post-COVID-19 condition by a Delphi consensus. Lancet Infect. Dis..

[26] Vandenbroucke J.P., von Elm E., Altman D.G., Gøtzsche P.C., Mulrow C.D., Pocock S.J., Poole C., Schlesselman J.J., Egger M. (2014). Strengthening the Reporting of Observational Studies in Epidemiology (STROBE): Explanation and elaboration. Int. J. Surg..

[27] EFSA Panel on Dietetic Products, Nutrition, and Allergies (NDA) (2010). Scientific Opinion on Dietary Reference Values for carbohydrates and dietary fibre. EFSA J..

[28] EFSA Panel on Dietetic Products, Nutrition, and Allergies (NDA) (2010). Scientific Opinion on Dietary Reference Values for fats, including saturated fatty acids, polyunsaturated fatty acids, monounsaturated fatty acids, trans fatty acids, and cholesterol. EFSA J..

[29] Aranceta Bartrina J., Arija Val V., Maíz Aldalur E., Martínez de la Victoria Muñoz E., Ortega Anta R.M., Pérez-Rodrigo C., Quiles Izquierdo J., Rodríguez Martín A., Román Viñas B., Salvador Castell G. (2016). Dietary guidelines for the Spanish population (SENC, December 2016); the new graphic icon of healthy nutrition. Nutr. Hosp..

[30] Gómez-Marcos M.A., Recio-Rodríguez J.I., Patino-Alonso M.C., Agudo-Conde C., Gómez-Sanchez L., Gómez-Sanchez M., Rodríguez-Sánchez E., García-Ortiz L. (2012). Protocol for measuring carotid intima-media thickness that best correlates with cardiovascular risk and target organ damage. Am. J. Hypertens..

[31] Van Bortel L.M., Laurent S., Boutouyrie P., Chowienczyk P., Cruickshank J.K., De Backer T., Filipovsky J., Huybrechts S., Mattace-Raso F.U., Protogerou A.D. (2012). Expert consensus document on the measurement of aortic stiffness in daily practice using carotid-femoral pulse wave velocity. J. Hypertens..

[32] Williams B., Mancia G., Spiering W., Agabiti Rosei E., Azizi M., Burnier M., Clement D., Coca A., De Simone G., Dominiczak A. (2018). 2018 Practice Guidelines for the management of arterial hypertension of the European Society of Hypertension and the European Society of Cardiology: ESH/ESC Task Force for the Management of Arterial Hypertension. J. Hypertens..

[33] (2010). The Reference Values for Arterial Stiffness’ Collaboration. Determinants of pulse wave velocity in healthy people and in the presence of cardiovascular risk factors: ‘establishing normal and reference values’. Eur. Heart J..

[34] London G.M., Pannier B., Safar M.E. (2019). Arterial Stiffness Gradient, Systemic Reflection Coefficient, and Pulsatile Pressure Wave Transmission in Essential Hypertension. Hypertension.

[35] Shirai K., Utino J., Otsuka K., Takata M. (2006). A novel blood pressure-independent arterial wall stiffness parameter; cardio-ankle vascular index (CAVI). J. Atheroscler. Thromb..

[36] Yamashina A., Tomiyama H., Takeda K., Tsuda H., Arai T., Hirose K., Koji Y., Hori S., Yamamoto Y. (2002). Validity, reproducibility, and clinical significance of noninvasive brachial-ankle pulse wave velocity measurement. Hypertens. Res..

[37] Takahashi K., Yamamoto T., Tsuda S., Maruyama M., Shirai K. (2020). The Background of Calculating CAVI: Lesson from the Discrepancy Between CAVI and CAVI(0). Vasc. Health Risk Manag..

[38] The World Health Organization (1988). MONICA Project (monitoring trends and determinants in cardiovascular disease): A major international collaboration. WHO MONICA Project Principal Investigators. J. Clin. Epidemiol..

[39] Schröder H., Fitó M., Estruch R., Martínez-González M.A., Corella D., Salas-Salvadó J., Lamuela-Raventós R., Ros E., Salaverría I., Fiol M. (2011). A short screener is valid for assessing Mediterranean diet adherence among older Spanish men and women. J. Nutr..

[40] Steeves J.A., Tyo B.M., Connolly C.P., Gregory D.A., Stark N.A., Bassett D.R. (2011). Validity and reliability of the Omron HJ-303 tri-axial accelerometer-based pedometer. J. Phys. Act. Health.

[41] Alonso J., Prieto L., Antó J.M. (1995). The Spanish version of the SF-36 Health Survey (the SF-36 health questionnaire): An instrument for measuring clinical results. Med. Clin..

[42] World Medical Association (2025). Declaration of Helsinki: Ethical Principles for Medical Research Involving Human Participants. JAMA.

[43] Koemel N.A., Senior A.M., Celermajer D.S., Grech A., Gill T.P., Simpson S.J., Raubenheimer D., Skilton M.R. (2023). Multi-Nutrient Analysis of Dietary Macronutrients with All-Cause, Cardiovascular, and Cancer Mortality: Data from NHANES 1999-2014. Nutrients.

[44] Song M., Fung T.T., Hu F.B., Willett W.C., Longo V.D., Chan A.T., Giovannucci E.L. (2016). Association of Animal and Plant Protein Intake with All-Cause and Cause-Specific Mortality. JAMA Intern. Med..

[45] Egert S., Amini A.M., Klug L., Kalotai N., Haardt J., Boeing H., Buyken A.E., Kroke A., Lorkowski S., Louis S. (2025). Protein intake and cardiovascular diseases: An umbrella review of systematic reviews for the evidence-based guideline on protein intake of the German Nutrition Society. Eur. J. Nutr..

[46] Naghshi S., Sadeghi O., Willett W.C., Esmaillzadeh A. (2020). Dietary intake of total, animal, and plant proteins and risk of all cause, cardiovascular, and cancer mortality: Systematic review and dose-response meta-analysis of prospective cohort studies. BMJ.

[47] Chen Z., Glisic M., Song M., Aliahmad H.A., Zhang X., Moumdjian A.C., Gonzalez-Jaramillo V., van der Schaft N., Bramer W.M., Ikram M.A. (2020). Dietary protein intake and all-cause and cause-specific mortality: Results from the Rotterdam Study and a meta-analysis of prospective cohort studies. Eur. J. Epidemiol..

[48] Libby P., Lüscher T. (2020). COVID-19 is, in the end, an endothelial disease. Eur. Heart J..

[49] Cicero A.F.G., Fogacci F., Desideri G., Grandi E., Rizzoli E., D’Addato S., Borghi C. (2019). Arterial Stiffness, Sugar-Sweetened Beverages and Fruits Intake in a Rural Population Sample: Data from the Brisighella Heart Study. Nutrients.

[50] Mozaffarian D. (2016). Dietary and Policy Priorities for Cardiovascular Disease, Diabetes, and Obesity: A Comprehensive Review. Circulation.

[51] Satoh N., Shimatsu A., Kato Y., Araki R., Koyama K., Okajima T., Tanabe M., Ooishi M., Kotani K., Ogawa Y. (2008). Evaluation of the cardio-ankle vascular index, a new indicator of arterial stiffness independent of blood pressure, in obesity and metabolic syndrome. Hypertens. Res..

[52] Gomaa A.A., Abdel-Wadood Y.A., Thabet R.H., Gomaa G.A. (2024). Pharmacological evaluation of vitamin D in COVID-19 and long COVID-19: Recent studies confirm clinical validation and highlight metformin to improve VDR sensitivity and efficacy. Inflammopharmacology.

[53] Santoro L., Zaccone V., Falsetti L., Ruggieri V., Danese M., Miro C., Di Giorgio A., Nesci A., D’Alessandro A., Moroncini G. (2023). Role of Endothelium in Cardiovascular Sequelae of Long COVID. Biomedicines.

[54] Xu S.W., Ilyas I., Weng J.P. (2023). Endothelial dysfunction in COVID-19: An overview of evidence, biomarkers, mechanisms and potential therapies. Acta Pharmacol. Sin..

